# A multidisciplinary primary care team consultation in a socio-economically deprived community: An exploratory randomised controlled trial

**DOI:** 10.1186/1472-6963-11-15

**Published:** 2011-01-24

**Authors:** Wai-Sun Chan, David L Whitford, Ronan Conroy, David Gibney, Brid Hollywood

**Affiliations:** 1 Division of Population Health Sciences, Royal College of Surgeons in Ireland, 120 St Stephens Green, Dublin 2, Ireland; 2 Family and Community Medicine, Royal College of Surgeons in Ireland-Medical University of Bahrain, PO Box 15503, Adliya, Kingdom of Bahrain; 3 Ballymun Primary Care Team, Ballymun Civic Centre, Ballymun, Dublin 11, Ireland

## Abstract

**Background:**

Psychosocial problems in socioeconomically deprived communities are not always amenable to traditional medical approaches. Mothers living in these areas are a particularly vulnerable group. The objective of this study was to evaluate the effectiveness of a lengthened multi-disciplinary team consultation in primary care in reducing anxiety and depression in mothers.

**Methods:**

This was a prospective randomised controlled trial of a multidisciplinary team consultation against normal care. 94 mothers were recruited from three general practices from an area of extreme socio-economic deprivation. Mothers randomised into the intervention group attended a multidisciplinary consultation with up to four case-specific health care professionals. Consultations addressed medical, psychological and social problems and lasted up to one hour. Conventional primary care continued to be available to the intervention families. Control group families received normal primary care services. The outcomes measured were anxiety and depression as using the Hospital Anxiety and Depression Scale (HADS), health status using SF36v2, and quality of life using the abbreviated Schedule for the Evaluation of Individual Quality of Life (SEIQoL-DW) at baseline, 6 months and 12 months.

**Results:**

Ordered logistic regression was used to analyse the data. There was no significant difference found between intervention and control groups after 6 months and 12 months in all of the measured outcomes.

**Conclusions:**

The new lengthened multi-disciplinary team consultation did not have any impact on the mental health, general health, and quality of life of mothers after 6 and 12 months. Other methods of primary health care delivery in socio-economically deprived communities need to be evaluated.

## Background

Individuals living in areas of socio-economic deprivation suffer poorer health [1]. They have a greater number of psychological problems, more long term illness, more multiple morbidity and more chronic health problems [2]. Current inequalities in health care further exacerbate this situation [3,4]. Primary and secondary care services in Ireland remain configured to give advantage to those with the least health need [5]. General practitioners in deprived areas work harder, with higher consultation rates and shorter consultation lengths than those in more affluent areas [6]. Unfortunately little of a concrete nature has been put in place to address this imbalance between health inequalities and access to health services and the basic principle of the inverse care law [7] still applies today [2,8].

The complex psycho-social problems presenting to general practitioners (GPs) in deprived areas are not always amenable to the traditional medical approach. Psychological distress is the most prevalent comorbidity in practices serving areas of high socio-economic deprivation [9,10]. Mothers of young children are particularly vulnerable to suffering psychological distress [11]. Lone mothers are also more likely to suffer with anxiety and depression than supported mothers, or non maternal groups of women [12]. This has been attributed to problems associated with financial hardship, poor social support and poor self-esteem experienced by lone mothers in comparison to their counterparts [13]. It is conceivable that these complex problems may benefit from a multi-disciplinary team approach. There are many studies which comment on the desirability of collaborative care between primary care and mental health services from the perspective of both the service user and provider [14]. Collaboration with specialist mental health services has yielded positive results in the treatment of common mental health disorders with some improvements in patients' psychopathology and quality of life over time [15]. However little has been done to look at the effectiveness of a multi-disciplinary team approach in the primary care context.

Several reviews have shown inconsistent conclusions on the benefit of longer consultation times in general practice [16-19]. However, studies have shown that the accurate diagnosis of psychological problems is associated with longer consultations [20] and that patients with psychological distress receive longer consultations [9,21]. The provision of longer consulting times for complex consultations in areas of deprivation increases patient enablement [22].

We designed a novel way of delivering primary care in a socio-economically disadvantaged community, involving a lengthened team based consultation. The objective of this trial was to evaluate the effectiveness of a lengthened multi-disciplinary team consultation in primary care in reducing anxiety and depression in mothers living in areas of socio-economic deprivation compared with usual care.

## Methods

### Setting

This study was set in three general practices in Ballymun, a suburb of North Dublin and an area of extreme socio-economic deprivation (HASSE deprivation index of 10, the highest ranking) [23]. The practice population has a disproportionately high proportion of females aged 20 to 49 and 46% of children are brought up in lone parent families [24].

The three practices involved in this study in Ballymun formed one of ten Primary Care Implementation Projects as part of the Irish government's health strategy in 2001 [25]. The new integrated primary care team consisted of the principal GPs (four), practice nurses, public health nurses, physiotherapists, an occupational therapist, psychiatric nurse, psychologist, dietician and social welfare officer. This study was planned and commenced during the formation of this team [26].

### Participants and recruitment

Eligible mothers were identified by their GPs and invited to participate during the course of their normal consultations. Eligible mothers were those who:

• had attended with personal or family psychological distress or problems relating to their social circumstances during the recruitment period.

and

• possessed a General Medical Service (GMS) card. This card is held by 28% of the Irish population, is means tested according to low weekly income and provides access to free medical care in Ireland [27]. Over 90% of patients served by Ballymun Primary Care Team possess a GMS card.

Excluded from the study were women under the age of 18 years, and women who had a learning disability or form of dementia.

The women who expressed interest in participation to their GPs were contacted by a researcher to arrange a meeting. During this meeting, the mothers were given information on the study and written consent was obtained by the researcher. The lead researcher (WC) then randomised the mothers into two equal sized non-stratified groups, using the method of sequentially numbered, opaque sealed envelopes (SNOSE) [28]. Here the numbered contents of these identical envelopes reveal the study allocation of the participation.

### The Intervention

Mothers allocated to the intervention group attended a multidisciplinary team consultation in addition to their usual care. These consultations took place in a phased manner to protect against major service disruption. Prior to the consultation the mothers and their problems were discussed openly at weekly Primary Health Care Team (PHCT) meetings in order to gain maximal input and advice. During these discussions it was decided which PHCT members (up to four) were most suited to attend the participants' consultation. A time and date for the consultation was also arranged to suit all participating in the trial consultation. Mothers were encouraged to bring a close relative or friend for support.

The PHCT member who knew the woman best, often the GP, chaired each trial consultation. They opened the consultation and facilitated the other team members to contribute in a controlled fashion. The consultation followed the mother's agenda and all areas of health and social functioning were explored. Each team member would contribute and offer advice around their field of expertise concerning the problems that arose in each case. At the end of each consultation a management plan was made and agreed with the mother and her family. Up to an hour was allowed for each consultation. The health professionals involved in the consultation would provide their services if applicable and continue to follow up the mothers and their families. Weekly team meetings assured the coordination of care plan.

#### Usual Care

Mothers who were allocated to the control group continued to receive their usual care from the PHCT. Usual care can vary between different healthcare providers [29]. Usual care in the Ballymun PCHT consisted of routine general medical care provided by the GPs. If a patient required a specialist opinion from a member of the PHCT, they needed a referral by their GP. The specialist would arrange an appointment with the patient in accordance with their waiting list before dealing with the patient's problem. The resources of this PHCT are not typical of primary care providers in Ireland as access for such services are normally only accessible through secondary care.

### Data, data collection and analysis

The primary outcome of this study was the psychological health of mothers as measured by the Hospital Anxiety and Depression Scale (HADS) [30]. HADS has been shown to perform well in assessing the severity of anxiety disorders and depression in primary care patients and the general population. Each HADS subscale for anxiety and depression is scored out of a maximum of 21points, with 0 - 7 representing normality, 8-10 mild disorder,11 - 14 a moderate disorder and 14 - 21 a severe disorder. Secondary outcomes involved assessing the mothers' health status using the SF36v2 questionnaire [31] and perspective on their quality of life using the schedule for the evaluation of individual quality of life- direct weighting (SEIQoL-DW) [32]. The SEIQoL-DW is a tool that allows the respondent to nominate areas in their life which are most important, rate their level of function or satisfaction with each, and indicate the relative importance of each to their overall quality of life. A global quality of life score is calculated from this. These three questionnaires were administered by research assistants to all participants at the time of recruitment in their home, and repeated again at 6 months and 12 months in the same manner.

Baseline data was collected from all study participants using questionnaires, GP computer records, and from the records of multidisciplinary team clinical meetings.

The sample size was calculated to give the study 90% power to detect a difference of 0.7 standard deviations, corresponding to a 75% probability that a person in the intervention group would do better than a person in the control group. This measure of effect size is often called the "common language effect size"[33], though it is, in fact, the parameter that underlies the Wilcoxon Mann-Whitney test[34]. The 75% effect size represents the minimum clinically important effect to be detected. However the outcomes used are not distributed normally, necessitating an adjustment to the sample size based on the relative power of the Wilcoxon test and the t-test (86%). Therefore the final sample size was calculated by multiplying the parametric sample size by 1.16. This gave a final sample size of 88 mothers in order to achieve 90% power at a 5% level of significance. 94 mothers were recruited to allow for drop outs.

All the data was stored in SPSS 14.0 statistical package and simple comparative tests were used to analyse the data for demographic and lifestyle baselines. Ordered logistic regression was used to measure the significant tests in which participants' baseline score was used as covariate. This adjusted the comparison between intervention and control groups for differences in baseline scores. Ordered logistic regression was used for the continuous scaled variables because these scales are ordinal, despite being rated on numeric scales. Ordinal logistic regression also provides a more tolerant test of hypotheses on such data, as it does not assume that the scale points are equidistant, or that errors are normal.

This randomised trial followed CONSORT guidelines [35]. However the methodology does deviate in that selection of participants was performed by members of the PHCT who were also delivering the intervention. Also the nature of both recruitment and the intervention meant blinding participants and the PHCT was not possible. However, the researcher assistant collecting data was blinded to participant allocation throughout the study. Ethical approval was granted through the Royal College of Surgeons in Ireland (Ref: REC2004/115).

## Results

**Figure 1:**
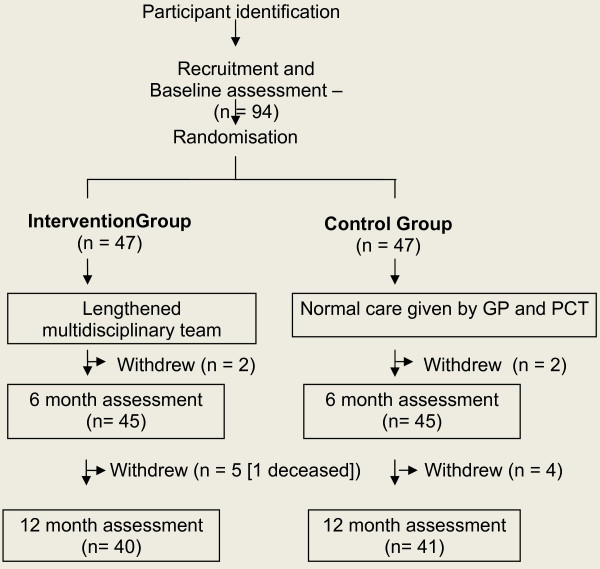
**Flow of patients through the study**.

Recruitment of participants and the study occurred between March 2005 and February 2006. Unfortunately no records were kept by the GPs as to how many mothers were approached to participate nor if any refused as invitations were made during the consultations with these patients. All ninety-four mothers who had expressed to GPs their interest to participate were recruited to the study. At the end of the study 81 mothers remained (Figure 1). Participants who withdrew stated time pressures and loss of interest as their reasons. One participant died during the study from non mental health reasons. Baseline characteristics of mothers in the intervention and control groups were comparable (Table 1). Each of the forty seven mothers in the intervention group participated in one trial consultation. Participation amongst PHCT members ranged from three to 31 consultations. Apart from the GPs, the public health nurses, social worker, home help and family support worker attended most frequently, indicating the types of problems presented by the mothers of these families. Participants were re-interviewed as near as possible to the 6 month and 12 month interval from baseline, with the time lag varying between zero to two weeks.

**Table 1:** Characteristics of participants at baseline

Characteristics		Control Group	Intervention Group**
**Mean age **(Standard Deviation, SD)		32 (7.79)	32 (5.76)

**Marital Status**	single	23 (49%)	25 (53%)
	married/with partner	15 (32%)	18 (38%)
	separated	7 (15%)	4 (9%)
	divorced	1 (2%)	0
	widowed	1 (2%)	0

**Number of children **Mean (SD)		3.17 (1.52)	3.04 (1.58)

**Employment**	Part time	4 (9%)	4 (9%)
	Unemployed	36 (77%)	39 (83%)
	Education	7 (14%)	4 (8%)

**Accommodation**	Owned	22 (49%)	12 (26%)
	Rented	24 (51%)	35 (74%)

**Number of bedrooms **Mean (SD)		2.96 (0.62)	2.60 (0.77)

**Number living in same accommodation **Mean (SD)		4.72 (1.66)	4.23 (1.57)

**Smoking status**	Smoker	34 (72%)	35 (74%)
	Non Smoker	13 (28%)	12(26%)

**Alcohol Consumption **(units)	Drinkers	29 (62%)	34(72%)
	Non-drinkers	18 (38%)	13(28%)

**Amount of alcohol**Consumed mean units (SD)		13.93 (12.88)	12.15(8.58)

**Substance abuse**	No history of substance	36 (77%)	38(81%)
	In treatment (methadone)	7 (15%)	5(11%)
	Treatment completed successfully (methadone)	0	1(2%)
	Still using drugs -Cocaine, cannabis, other (not disclosed)	4(8%)	3(6%)

**Outcome Measures **Mean (Standard deviation)			

**HADS ^† ^Depression subscale**		7.7 (4.1)	8.3(4.6)
**HADS Anxiety subscale**		10.8 (4.6)	11.4(3.8)
**HADS Total score**		18.3 (7.9)	19.9(7.6)
SF36 * Physical Health Summary Score		47.9 (9.3)	46(10.34)
**SF 36 Mental Health Summary Score**		33.7 (15.9)	33.2(13.13)
**SEIQoL ^Δ ^Global Score**		44.6 (26.1)	42.9(22.8)

** There were no significant differences between the control and intervention groups at baseline (p > 0.05)

^† ^HADS contains 14 questions and consists of two subscales: anxiety and depression. Each question is rated on a four-point scale, giving maximum scores of 21 for anxiety and depression. Scores of 0 - 7 represent normality, 8-10 borderline disorder and 11 or more represents a significant 'case' of psychological morbidity.

* SF-36 scores are scored on a range of 0 (poorest health) - 100 (best health possible).

^Δ ^SEIQoL global score is calculated by multiplying the individual's current self rating on each cue by the corresponding cue weight and summing the products across the five cues. This global quality of life score can range from 0 to 100

### Outcome measures

The primary outcome of this study was HADS scores of the participants. 57% of mothers scored as having moderate to severe anxiety (scoring more than 11 points on a scale out of 21), and 30% scored as suffering with moderate to severe depression at baseline. Also 23% of mothers scored as having both moderate to severe anxiety and depression at baseline.

Table 2 shows the scores for the primary and secondary outcomes at 6 months and 12 months for both intervention and control groups. This reveals that the intervention had no effect on the total HADS score compared with the control at 6 months and 12 months. Significance was detected in the HADS depression subscale at 6 months between the two groups (p = 0.038) but was not seen at 12 months.

**Table 2:** Outcome Measures at 6 Months and 12 Months and adjusted differences between intervention and control groups

	Control Mean	SD	Intervention mean	SD	z	Sig
**6 months**						

HADS total score	17.5	9.8	21.5	8.7	-1.880	0.061

HADS D	6.8	5.3	9.0	4.9	2.070	0.038

HADS A	10.8	5.3	12.5	4.9	1.330	0.183

						

SF-36 Physical	46.5	10.0	46.0	9.6	0.440	0.660

SF-36 Role limitation physical	44.3	10.9	43.0	12.4	0.690	0.493

SF-36 Bodily pain	46.2	12.2	42.2	13.1	-1.420	0.157

SF-36 General health	42.4	11.2	39.0	14.3	-1.320	0.188

SF-36 Vitality	40.8	14.9	39.6	13.8	-0.420	0.671

SF-36 Social functioning	39.9	14.7	33.0	13.7	-2.160	0.031

SF-36 Role limitation emotional	39.6	14.2	36.7	13.0	-0.880	0.379

SF-36 Mental health	39.5	16.0	34.1	14.8	-1.900	0.057

SF-36 Physical health summary score	47.6	9.2	46.5	12.0	0.490	0.627

SF-36 Mental health summary score	37.8	15.8	32.3	14.7	-1.880	0.061

						

SEIQoL score	41.9	26.8	33.5	21.1	-1.460	0.145

**12 Months**						

HADS total score	17.9	9.3	19.5	9.3	0.160	0.871

HADS D	6.9	4.9	8.3	5.0	0.840	0.400

HADS A	11.0	5.2	11.2	5.1	-0.660	0.508

						

SF-36 Physical	47.5	9.4	45.5	9.2	-0.940	0.348

SF-36 Role limitation physical	42.9	12.7	41.5	12.3	0.250	0.801

SF-36 Bodily pain	43.2	13.1	39.2	13.3	-1.190	0.234

SF-36 General health	43.3	11.9	39.1	14.0	-0.760	0.449

SF-36 Vitality	41.6	12.1	38.8	12.4	-1.170	0.243

SF-36 Social functioning	37.5	14.2	36.5	15.6	0.050	0.963

SF-36 Role limitation emotional	36.6	14.3	35.3	13.8	0.080	0.939

SF-36 Mental health	37.1	14.3	36.6	14.1	0.130	0.893

SF-36 Physical health summary score	47.9	10.5	44.6	11.6	-0.660	0.507

SF-36 Mental health summary score	34.9	14.6	34.3	15.7	-0.180	0.855

						

SEIQoL score	43.8	24.2	43.1	25.2	0.130	0.899

#Values adjusted for baseline scores

^† ^HADS contains 14 questions and consists of two subscales: anxiety and depression. Each question is rated on a four-point scale, giving maximum scores of 21 for anxiety and depression. Scores of 0 - 7 represent normality, 8 - 10 borderline disorder and 11 or more represents a significant 'case' of psychological morbidity.

* SF-36 scores are scored on a range of 0 (poorest health) - 100 (best health possible).

^Δ^SEIQoL global score is calculated by multiplying the individual's current self rating on each cue by the corresponding cue weight and summing the products across the five cues. This global quality of life score can range from 0 to 100

The intervention also had no effect on the summary scores of the SF36-v2 at both 6 and 12 months, although some significance was detected for social functioning alone at 6 months. The SEIQoL-DW tool assessed each mothers' perspective on their quality of life. The most popular cues nominated as being most important to their quality of life concerned their local environment, their own mental health, finances and their family (Table 3). The intervention also had no significant impact on the global score for quality of life using SEIQoL-DW at either 6 months or 12 months.

**Table 3:** Key quality of life cues as nominated by participants in their SEIQoL-DW and their frequency

SEIQoL cues	Number of times nominated
	(Total nominations = 470)

Mental health	54

Local Area and facilities	54

Family health	45

Family relationships	41

Physical health	29

Financial	28

Exercise/leisure/social activities	22

Children's' education	21

Energy levels	20

Sleep/Rest	20

Job/Work/Study	19

Overall personal health	18

Loneliness	17

## Discussion

### Summary of main findings

This exploratory study represents the first attempt to evaluate a team based consultation model in primary care. The study shows that implementation of a consultation model offering the elements of more time for the patient and provision of the expertise of case specific members of a primary care team did not have any effect on anxiety and depression in mothers from a socio-economically deprived area. Interestingly those in the intervention group were found to be significantly more depressed than the control group after 6 months as detected by HADS depression subscale. The effect brought about by focusing on the gravity of this group's psychosocial problems in the lengthened team consultation might be an explanation for this finding. The intervention also had no effect on the mothers' health status nor their perceived quality of life. The participants in this study had high levels of anxiety and depression, and poor quality of life scores. It is therefore unlikely that the lack of effect was due to poor identification of at-risk mothers.

In line with evidence of the beneficial effects of lengthened consultations [12-14,16-18], it seems probable that the lengthened multi-disciplinary team consultation allowed for better recognition and assessment of the health and psychosocial problems of these mothers. However it is possible that the intervention was not intensive enough to bring about change in this vulnerable group. There are so many social, personal and environmental determinants of mental and physical health that are beyond the scope of the intervention that it may have been surprising for it to have a measurable effect. These participants might have benefited from further lengthened multidisciplinary consultations and more rigorous follow up from health professionals to manage and review the problems that had been identified.

A further explanation for the negative results might have resulted from contamination in patient management. Some of the strategies and solutions offered during the multidisciplinary consultation to some participants might have given the health professionals in this PCHT ideas about how to approach new patients as well as those in the control group if they were involved in their care. On reflection, a cluster randomised controlled trial at the level of the GP practice may have resolved this.

The power calculation for this study required there to be 88 mothers randomised and so with only 81 mothers remaining after 12 months interpretation of the results is difficult. However our findings were also negative after only 6 months when there were still an adequate number of patients in follow-up. Although social functioning scored significantly worse after 6 months in the intervention group, one significant finding is not altogether surprising given the number of tests conducted and this is unlikely to be of clinical significance.

### Strengths and limitations of the study

Identification of mothers and their families took place during regular consultations by the doctors in this study. It is acknowledged that in a pragmatic study of this kind there was a potential for a selection bias to be introduced. Another limitation is that the numbers of women approached by GPs and the number who refused are unknown. The families recruited may have been those who needed the most help biasing the results towards the null hypothesis, or alternatively families with the greatest need may have declined participation.

Although patient randomisation did ensure that at least both intervention and control groups were comparable in their characteristics, there may have been inconsistencies within the intervention itself. Variations in team configurations for each consultation may have led to variations in approach for each of the consultations, which would have been difficult to control for. Another feature which would have been difficult to control for would be medication, which may have been prescribed during the course of the study. Whilst prescribing for mild to moderate anxiety or depression may not be recommended [36], we cannot assume that none of our participants received any medication and hence not collecting data on the medications of participants is an omission of the study.

In the absence of a perfect study tool to assess the outcomes of mental health in mothers, HADS was chosen as it is validated in primary care. The sensitivity of HADS to detect differences between the groups in a study such as this is, however, not clear. A more rigorous selection or development of research tools in the planning stage may have given more accurate measurements of our desired outcomes.

### Comparison with existing literature

Although there are no published studies of evaluation of multidisciplinary primary health care team consultations, there have been several studies evaluating the role of mental health workers in primary care and their effect on anxiety and depression. A study on the effect of a consultation with a mental health worker found no difference in service utilisation and costs between those who received consultations and those who did not [37]. Even when studies have found beneficial effects on mental and social functioning in the short term, these benefits have dissipated by 12 months [38]. Reviews of counselling in primary care also indicate that beneficial effects in patients with common mental health disorders are often 'short term' [39]. A study evaluating a multidisciplinary consultation for frequent attenders found that patients' medical costs and GP visits fell within the 12 months following the intervention [40] but did not look at clinical outcomes.

### Implications for future research or clinical practice

The results do not support the full implementation of a lengthened multi-disciplinary team consultation model to mothers with psychosocial problems. Although not measured, much time and effort was expended in order to run this consultation model. Whilst pragmatically it appears that those who received the intervention received very good care, it appears our measures were unable to detect this due to the demands of such a deprived population. Measures for outcomes including patient satisfaction, problem recognition and team building were not used. However a qualitative assessment has been carried out in order to assess these.

A more intensive approach of the lengthened multi-disciplinary team consultation at regular intervals per patient may have more effect on this study population. Also it may be more effective to limit this intervention to those who actually had moderate to severe anxiety or depression according to HADS at baseline. Another approach may be to build and develop stronger collaboration with the mental health service which has proven positive in other studies in other countries [14,15] and add this to the trial consultation. Further studies are needed to explore this difficult problem in this deprived population.

## Conclusions

A lengthened multi-disciplinary team consultation did not have any impact on the mental health, general health, and quality of life of mothers after 6 and 12 months. Other methods of primary health care delivery in socio-economically deprived communities need to be evaluated.

## Competing interests

The authors declare that they have no competing interests.

## Authors' contributions

WSC, DLW, DG and BH participated in the conception and design of the study. WSC drafted the manuscript which was critically reviewed and revised by DLW. RC provided statistical advice, performed statistical analysis and critically reviewed the draft. WSC, DG and BH coordinated the study in the health centres. All authors read and approved the final manuscript.

## Pre-publication history

The pre-publication history for this paper can be accessed here:

http://www.biomedcentral.com/1472-6963/11/15/prepub
